# The macrophage C-type lectin receptor CLEC5A (MDL-1) expression is associated with early plaque progression and promotes macrophage survival

**DOI:** 10.1186/s12967-017-1336-z

**Published:** 2017-11-10

**Authors:** Weixin Xiong, Haibo Wang, Lin Lu, Rui Xi, Fang Wang, Gang Gu, Rong Tao

**Affiliations:** 1 Department of Cardiology, Ruijin Hospital, Jiao Tong University School of Medicine, No. 197, Ruijin Er Road, Huangpu District, Shanghai, 200025 China; 2 Institute of Cardiovascular Disease, Jiao Tong University School of Medicine, Shanghai, 200025 China

**Keywords:** Atherosclerosis, Plaque progression, Macrophage, Apoptosis, Myeloid DAP12-associating lectin-1

## Abstract

**Background:**

Biomarkers of early plaque progression are still elusive. Myeloid DAP12-associating lectin-1 (MDL-1), also called CLEC5A, is a C-type lectin receptor implicated in the progression of multiple acute and chronic inflammatory diseases. However, the relationship between its level and atherosclerosis is unknown. In this study, we aimed to investigate the correlation between macrophage MDL-1 expression and early atherosclerosis progression.

**Methods:**

Immunofluorescence staining, real-time PCR and western blot were performed to analyze MDL-1 expression in aorta or mice macrophages. The role of MDL-1 in macrophage survival was further investigated by adenovirus infection and TUNEL assay.

**Results:**

Significant MDL-1 expression was found in advanced human and apoE−/− mice atherosclerotic plaques, especially in lesional macrophages. In the model of atherosclerosis regression, we found MDL-1 expression was highly downregulated in lesional macrophages from ldlr−/− mouse regressive plaques, coincident with a reduction in lesional macrophage content and marker of M1 proinflammatory macrophages. Furthermore, we found MDL-1 was significantly expressed in inflammatory M1 subtype polarized bone marrow-derived macrophages. In vitro experiments, the level of MDL-1 was remarkably elevated in macrophages treated with pathophysiological drivers of plaque progression, such as oxidized low-density lipoprotein (ox-LDL) and hypoxia. Mechanistically, we demonstrated that MDL-1 overexpression notably promoted macrophage survival and decreased cleaved caspase-3 expression under ox-LDL stimulation, which suggested that it could maintain lesional macrophage survival and cause its accumulation.

**Conclusions:**

This study firstly demonstrated that MDL-1 is mainly expressed in atherosclerotic lesional macrophages and increased macrophage MDL-1 expression is associated with early plaque progression and promotes macrophage survival.

**Electronic supplementary material:**

The online version of this article (10.1186/s12967-017-1336-z) contains supplementary material, which is available to authorized users.

## Background

Atherosclerosis is both a metabolic and an immunoinflammatory disease [1]. Either disturbance of lipid level or immune system balance might contribute to atherogenesis [2, 3]. The role of inflammatory cells, especially monocyte/macrophage, is central in the disease and their accumulation is relevant to plaque development and also a major contributor to the persistence of disease state [4, 5]. In the early phase of atherosclerosis, monocytes recruit and transmigrate to the subendothelial layer of the aortic wall and differentiate into macrophages. These inflammatory macrophages could then either emigrate from the site of localized inflammation or undergo apoptosis process, which reduces plaque burden and causes atherosclerosis regression [6, 7]. However, atherosclerotic inflammation is not readily resolved and these macrophages become foam cells persisting in the arterial wall, which leads to plaque instability and disease progression. Therefore, finding the target and biomarker of lesional macrophage retention and accumulation is promising for prevention of later adverse cardiovascular outcome occurrence.

Myeloid DAP12-associated lectin-1 (MDL-1) is also called C-type lectin domain family 5-member A (CLEC5A), which has a short cytoplasmic region lacking particular signaling motifs and associates noncovalently with DAP-12 to transmit downstream signaling via DAP-12 immuno-receptor tyrosine-based activation motif (ITAM) [8]. The endogenous ligand for MDL-1 has yet to be found and dengue virus particle is the only ligand identified in immune cells [9]. Recent researches showed that MDL-1 is expressed on mouse neutrophils, monocytes/macrophages and human peripheral blood monocytes as well as myeloid cell precursors [9, 10]. Studies also demonstrated a strong expression of MDL-1 and DAP-12 in mature and differentiated monocytes/macrophages in contrast to undifferentiated bone marrow CD34^+^ stem cells or monocytes [11]. Functionally, MDL-1 acts as a signaling receptor for proinflammatory cytokine release [12, 13] and is implicated in mediating multiple acute and chronic inflammatory diseases, including hemorrhagic fever, lethal shock, virus-induced brain damage, chronic obstructive pulmonary disease and development of autoimmune arthritis [14–18]. However, the correlation of MDL-1 with atherosclerosis and its behavior are still elusive and remain unknown. Moreover, investigations on 9P21.3 coronary artery disease (CAD) risk locus of human macrophages exhibited remarkable differences in gene expression including CCL-8, CCL-2 and MDL-1 between myocardial infarction patients and controls [19], which suggested macrophage MDL-1 might be associated with CAD progression. Therefore, in this study, we aim to study the relationship between macrophage MDL-1 expression and early atherosclerosis progression and its role in macrophage apoptosis.

Here, we showed that MDL-1 is abundantly expressed in macrophages of human and apoE−/− as well as ldlr−/− mice advanced atherosclerotic lesions and downregulated during early atherosclerotic plaque regressive phase. Moreover, we further found MDL-1 was highly expressed in M1 subtype macrophage and elevated when treated with pathophysiological drivers of plaque progression (e.g. ox-LDL or hypoxia). Mechanistically, we found MDL-1 overexpression notably inhibited ox-LDL-induced macrophage apoptosis and amplified monocyte chemotactic protein-1 (MCP-1) production, thereby implicating that it might maintain lesional macrophage survival and link abnormal hyperlipidemic environment to macrophage accumulation during early atherosclerotic plaque progression.

## Methods

### Clinical samples

Human femoral arteries with atherosclerotic plaques were obtained from three patients who underwent leg amputation surgery, and the arteries were then embedded in paraffin. The internal thoracic arteries (n = 3) were obtained as normal artery. Baseline demographics, risk factors for atherosclerosis, clinical biochemical results and medications were recorded. The study protocol was approved by the Ethics Committee of Ruijin Hospital, Shanghai Jiaotong University School of Medicine, and written informed consent was obtained from all patients. Since the study did not involve any intervention, it was not prospectively registered.

### Isolation of human peripheral blood monocytes (PBMCs)

We obtained samples of peripheral blood from significant CAD patients with stable angina (n = 3) who had at least a major epicardial coronary artery luminal diameter narrowing ≥ 50% observed in coronary angiography examination and healthy volunteers (n = 3). We then isolated PBMCs using dextran sedimentation and a Ficoll–Hypaque density-gradient separation and enriched by a magnetically negative depletion protocol (Miltenyi Biotec) as previously described [20]. Purified PBMCs were lysed in a lysis buffer and a protease inhibitor cocktail and stored at − 80 °C until use.

### Experimental animals and diet

All animal experiments were conducted according to the experimental protocols approved by the Committee on Animal Resources of Shanghai Jiaotong University. 6-week old apoE−/− and ldlr−/− mice were purchased from Model Animal Research Center of Nanjing University and maintained in a pathogen-free facility in the Animal Experiment Center of Ruijin Hospital, Shanghai Jiaotong University School of Medicine. All apoE−/− mice were fed a chow diet for 2 weeks and half of them were then changed into a high-fat diet (HFD, 21% fat, 0.2% cholesterol, 23% protein, and 40.5% carbohydrate; Research Diets, Inc.) for 8 weeks before sacrifice. In the model of regression, 8-week old ldlr−/− mice were placed on a HFD for 8 weeks and were either sacrificed (baseline) or switched to chow diet for 4 weeks as described [21]. Aortic and heart tissues were then exposed and obtained. Blood samples and serum were collected both in baseline and plaque regression ldlr−/− mice group. Cholesterol levels were measured by HDL and LDL/VLDL cholesterol assay kit (Abcam) according to the manufacturer’s protocol.

### Histology and immunostaining

Serial frozen sections of gelatin permeabilized aortic sinus lesions were prepared from OCT embedded heart tissue using a microtome as described [22]. For lesion immunostaining, 10 μm sections were blocked with 10% normal serum for 1 h and then incubated with primary antibodies recognizing MDL-1 (1:50 dilution, abcam), CD68 (1:100 dilution, AbD Serotec), MOMA-2 (Rat anti-mouse MOMA-2, 1:50 dilution, AbD Serotec), DAP-12 (1:50 dilution, Santa Cruz), Ly-6G (1:100 dilution, AbD Serotec), iNOS (1:100 dilution, BD biosciences) or Arg-1 (1:100 dilution, BD biosciences) overnight at 4 °C. All sections were washed and incubated with fluorescent secondary antibodies (1:1000 dilution, invitrogen) for 60 min. Sections without primary antibody served as negative controls. Finally, all the sections were washed in TBS and mounted in glycerol-based mounting medium containing antifade reagent with DAPI (Invitrogen) and then photomicrographed and digitized with an Olympus DP50-CU digital camera mounted on an Olympus BX51 microscope. Lesional MDL-1 positive macrophages were determined as the percentage of MDL-1 and MOMA-2 double positive macrophages per lesional area. All immunostaining images were analyzed with the NIH Image-Pro Plus software (Media Cybernetics, Bethesda, MD).

### Cell culture

Bone marrow-derived macrophages (BMDMs) were prepared from mice bone marrow and resuspended and cultured in RPMI 1640 medium (Gibco) supplemented with 10 ng/mL monocyte-colony stimulating factor (M-CSF, R&D systems, Minneapolis, MN, USA), 10% heat-inactivated fetal bovine serum (FBS), 50 U/mL penicillin and 50 U/mL streptomycin as described [23]. For macrophage subtype polarization, we changed to fresh stimulation medium: for M1 activation, use RPMI 1640 containing 10% FBS and 100 ng/mL LPS (Sigma Aldrich); for M2 activation, use RPMI 1640 containing 10% FBS with 10 ng/mL IL-4 (Peprotech). Peritoneal primary macrophages were obtained from 6-week old C57BL/6 mice by peritoneal lavage 4 days after intraperitoneal injection of 3% thioglycollate (Sigma Aldrich). The cells were washed and resuspended and then cultured in RPMI 1640 medium supplemented with 10% heat-inactivated FBS, 50 U/mL penicillin and 50 U/mL streptomycin as previously described [24]. To study cellular MDL-1 expression, we maintained peritoneal macrophages to subconfluence and stimulated them with 50 μg/mL ox-LDL/LDL (AbD Serotec) or CoCl_2_ (Sigma Aldrich) with or without pretreatment of 10 μg/mL HDL (Sigma Aldrich) or 100 μmol/L HIF-1α inhibitor (Calbiochem, EMD Millipore). LDL was purified to homogeneity by ultracentrifugation, oxidized using 20 μmol/L cupric sulphate in phosphate buffered saline (PBS) at 37 °C for 24 h before oxidation was terminated with ethylene diamine tetraacetic acid (EDTA). The level of oxidation was measured using thiobarbituric acid reactive (TBAR) determination according to a malondialdehyde standard. The migration factor of ox-LDL was 2.2. To assess MDL-1 cellular effects, we infected the macrophages with adenoviral vector overexpressing MDL-1 before treatment with ox-LDL.

### Western blot

The concentrations of all tissue and cell lysate protein samples were examined according to the method of Bradford as described [25]. After normalizing for equal protein concentration, the protein samples were resuspended in SDS protein buffer before separation by SDS-PAGE. We then transferred the samples to polyvinylidene difluoride membranes. The membranes were probed with primary antibodies overnight at 4 °C and horseradish peroxidase-conjugated secondary antibodies for 60 min at room temperature. We then detected the protein bands using an electrochemiluminescence (ECL) system (GE Healthcare Biosciences, Pittsburgh, PA, USA). Densitometry analysis of the protein bands was carried out using ImageJ software from the National Institutes of Health (NIH).

### Quantitative real-time polymerase chain reaction (RT-PCR)

Total BMDM RNA was isolated using a Trizol reagent (Invitrogen, Carlsbad, CA, USA). For reverse transcription, we converted 1 ug of total RNA into first strand complementary DNA (cDNA) in a 20 μL reaction volume using a reverse transcription kit (Promega, Madison, WI, USA) according to the manufacturer’s instruction. Quantitative real-time PCR analysis was performed (StepOne, Applied Biosystems) using SYBR Green (Takara, Otsu, Japan). Gene expression levels were normalized with GAPDH, and data were analyzed with StepOne software v2.1 (Applied BioSystems). Following primers were used: 5′-TACCCCCAATGTGTCCGTC-3′ for mouse GAPDH-F and 5′- GGTCCTCAGTGTAGCCCAAG-3′ for mouse GAPDH-R. 5′-TCGGGGCTTATCGTAGTAGTG-3′ for mouse MDL-1-F and 5′-TGTAGGCATGGTACTTTCGTCAT-3′ for mouse MDL-1-R.

### Terminal deoxynucleotidyl transferase dUTP nick end labeling (TUNEL) staining

Macrophage apoptosis assessment was detected by TUNEL staining using the in situ cell death detection kit (Roche) according to the instruction. Briefly, adherent cell chamber slides were washed several times and fixed in freshly prepared 4% paraformaldehyde solution. We then incubated the slides in 0.1% Triton X-100 solution for 2 min on ice. After washed with PBS, the slides were incubated with TUNEL reaction mixture for 60 min at 37 °C in a humidified atmosphere in the dark. The slides were then washed and mounted in glycerol-based mounting medium containing antifade reagent with DAPI (invitrogen) and then photomicrographed and digitized with an Olympus DP50-CU digital camera mounted on an Olympus BX51 microscope. The average percent of TUNEL^+^ cells was determined as the percentage of TUNEL^+^ nuclei per field.

### Adenovirus infection

Freshly prepared peritoneal macrophages were seeded in 6-well dish and maintained in RPMI 1640 medium for 24 h. The recombinant adenovirus with or without MDL-1 overexpressed adenoviral vector (pAdeno-MCMV-EGFP-P2A-MDL1-3FLAG) was added to cell with MOI (multiplicity of infection) of 1000 plaque-forming units per cell in RPMI 1640 medium without FBS for 2 h. Infection was carried out with the indicated MOI for 48 h, after which the infection medium was aspirated and replaced with fresh complete medium. The ability of infected macrophages to express MDL-1 was assessed by Western blot analysis.

### Statistical analysis

Statistical analyses were performed using Student’s t test or ANOVA when data followed a normal distribution or Mann–Whitney U test when data did not follow a normal distribution, using the software SPSS 16.0 (SPSS Inc., Chicago, IL, USA). A p value below 0.05 was considered statistically significant.

## Results

### MDL-1 is mainly expressed in macrophages of advanced atherosclerotic plaques and its level decreases in regressive plaques

**Fig. 1:**
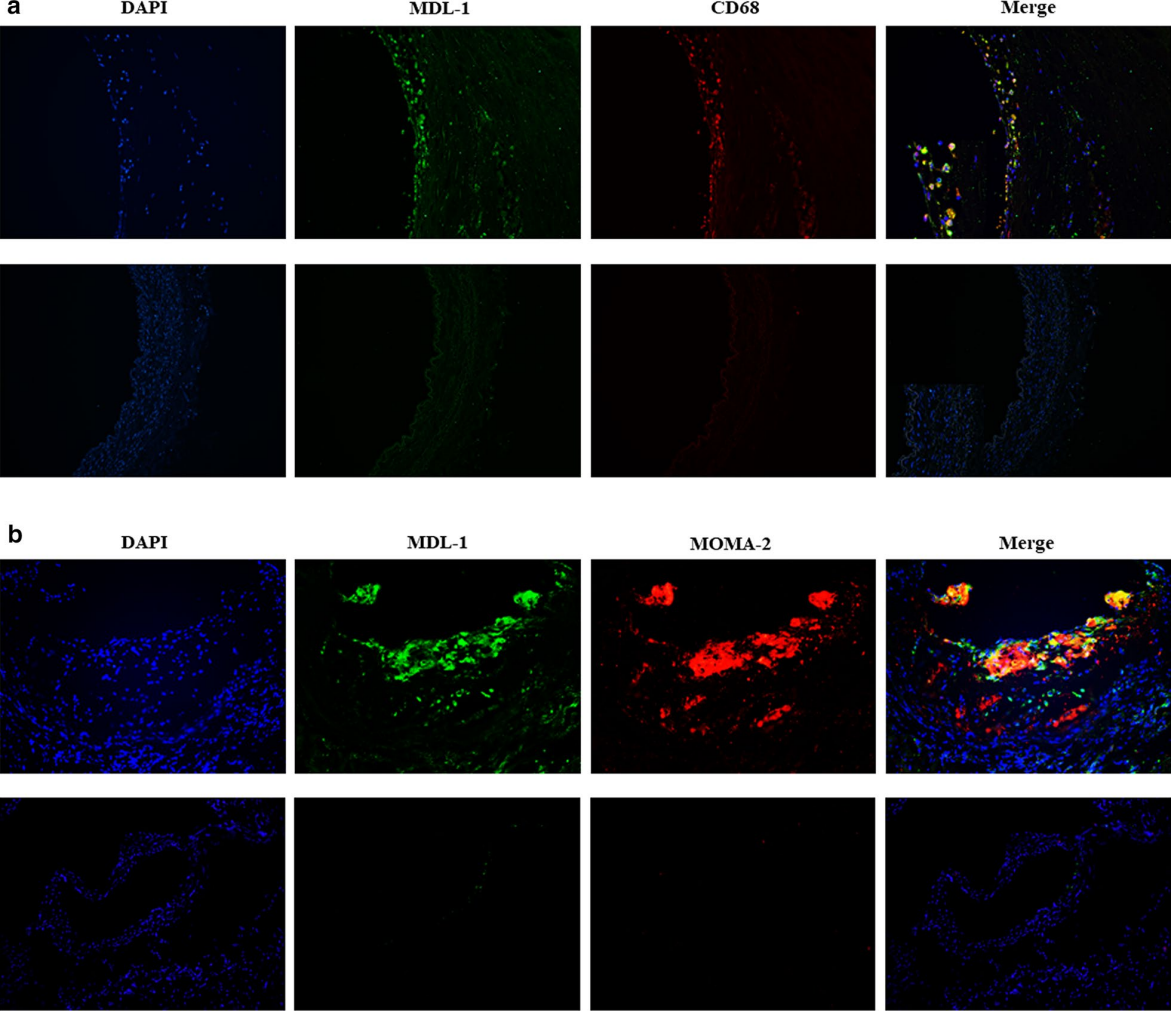
MDL-1 is mainly expressed by lesional macrophages in human and apoE−/− mice atherosclerotic plaques. **a** MDL-1 (green) or CD68 (red) were stained in human femoral arterial plaques (top panel) and normal internal thoracic arteries (bottom panel). Areas of colocalization are shown in yellow in the merged image. Images are representative of 3 subjects. **b** MDL-1 (green) or MOMA-2 (red) were stained in apoE−/− mice aortic root sections from western diet group (top panel) and chow diet group (bottom panel). Areas of colocalization are exhibited in yellow in the merged image. Images are representative of 3 mice

To demonstrate the macrophage expression of MDL-1 at protein level in atherosclerotic lesions, human femoral arteries with atherosclerotic plaques and internal thoracic arteries (as controls) were obtained and prepared (donor baseline characteristics in Table 1). In these arterial sections, we uncovered that MDL-1 was mainly colocalized with CD68 in advanced human atherosclerotic plaques, which indicates MDL-1 is abundantly expressed in lesional macrophages (Fig. 1a). This finding was also demonstrated in early advanced atheromata from apoE−/− mice fed a 8-week HFD, which exhibited significant colocalization with MOMA-2 (Fig. 1b). However, we did not find MDL-1 expression in normal control arterial sections (Fig. 1a, b). Furthermore, staining for MDL-1 adaptor DAP-12 also colocalized with MDL-1 in early advanced apoE−/− mice plaques (Additional file 1: Figure S1), which suggests MDL-1 associates with DAP-12 and plays its role on advanced atherosclerotic lesion. Since these MDL-1 expressed lesional macrophages derived from peripheral blood circulating monocytes and MDL-1 was also found to be expressed in monocytes, we detected MDL-1 expression in PBMCs from both CAD patients and healthy volunteers. As we expected, MDL-1 was demonstrated to be expressed in circulating monocytes from CAD patients, much higher than healthy controls (*p* < 0.01; Additional file 2: Figure S2), which showed increased MDL-1 expression in circulating monocytes correlated to atherosclerotic progression.

**Table 1:** Baseline characteristics and medical treatment of patients

Variables	Patients (n = 3)
Age (years)	60.7 ± 7.4
Male, n (%)	3 (100)
BMI (kg/m^2^)	27.3 ± 1.4
Smoking, n (%)	1 (33.3)
Hypertension, n (%)	3 (100)
Hyperlipidemia, n (%)	2 (66.7)
Leukocyte (10^9^/L)	6.0 ± 0.6
Platelet (10^9^/L)	197.3 ± 10.7
FBG (mmol/L)	5.5 ± 1.0
2h-PG (mmol/L)	10.5 ± 3.4
HbA1c (%)	7.0 ± 1.1
Blood urea nitrogen (mmol/L)	4.5 ± 0.3
Creatinine (μmol/L)	75.0 ± 6.7
Uric acid (mmol/L)	290.0 ± 37.6
hs-CRP (mg/L)	1.0 ± 0.5
Triglyceride (mmol/L)	1.2 ± 0.2
Total cholesterol (mmol/L)	4.7 ± 0.3
HDL-cholesterol (mmol/L)	1.1 ± 0.2
LDL-cholesterol (mmol/L)	3.2 ± 0.3
Lipoprotein (a) (g/L)	0.1 ± 0.03
Medications, n (%)	
Anti-platelet	3 (100)
Beta-blocker	2 (66.7)
ACE-I/ARB	2 (66.7)
Statin	3 (100)

*BMI* body mass index, *FBG* fasting blood glucose, *2h*-*PG* 2h-postprandial glucose, *hs*-*CRP* high sensitivity C-reactive protein, *HDL* high-density lipoprotein, *LDL* low-density lipoprotein, *ACE*-*I* angiotensin converting enzyme inhibitor, *ARB* angiotensin receptor blocker

**Fig. 2:**
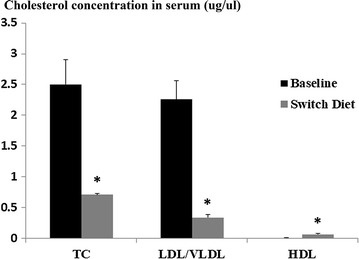
The serum total and LDL cholesterol levels were significantly lowered in LDLr−/− mice when switching to a chow diet. LDLr−/− mice fed a 8-week HFD were either sacrificed for baseline or switched to a chow diet for another 4 weeks. Total cholesterol and LDL/VLDL cholesterol concentration were decreased and HDL cholesterol was elevated in switching group compared with baseline group mice (n = 4–5 mice/group). *p < 0.05 compared with baseline group mice. *LDLr* low-density lipoprotein receptor, *HFD* high-fat diet, *LDL/VLDL* low-density lipoprotein/very low-density lipoprotein

**Fig. 3:**
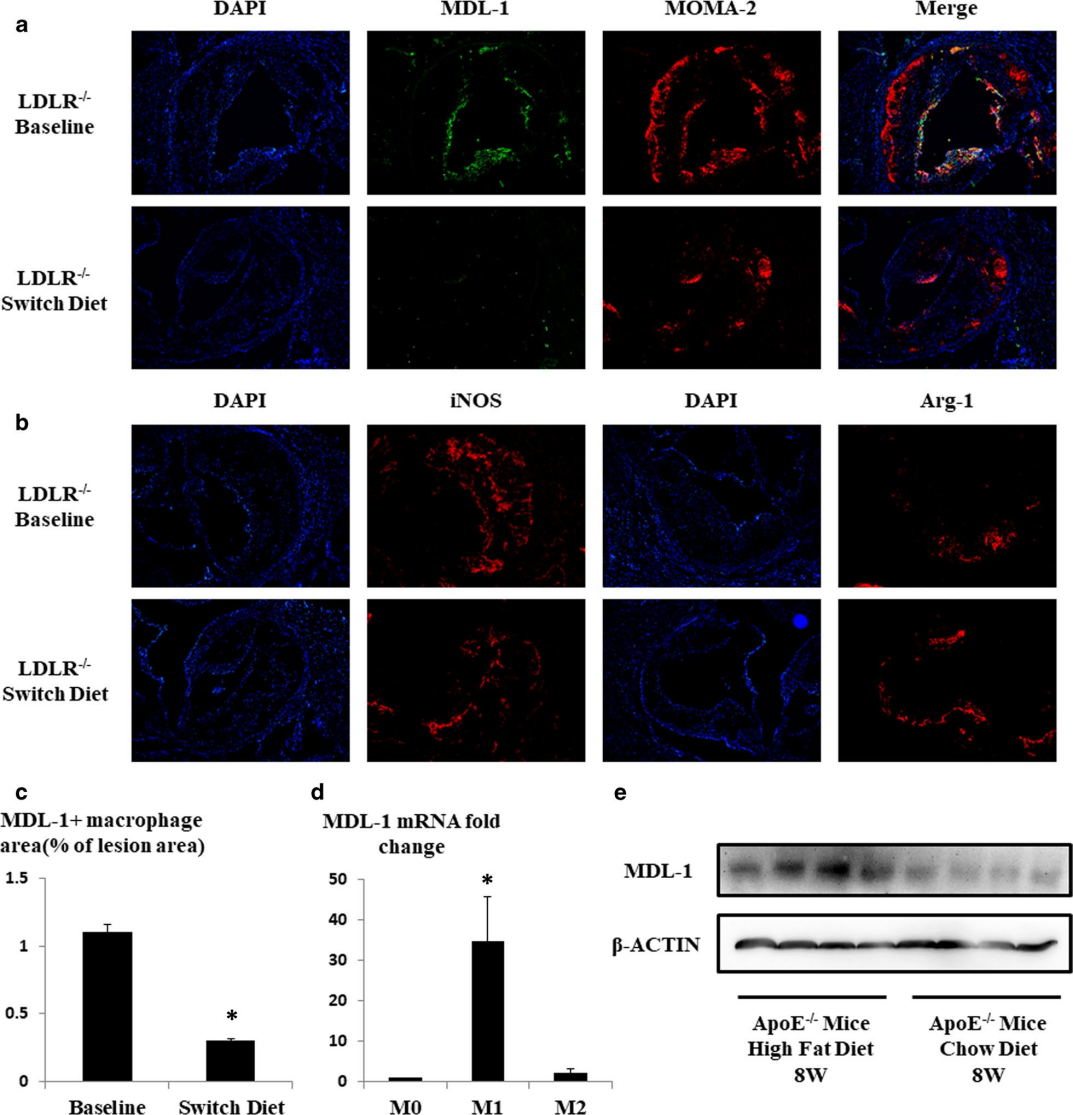
MDL-1 expression is downregulated in regressive plaques and has a proinflammatory phenotype in the lesion. **a** Immunofluorescent staining of MDL-1 (green) and MOMA-2 (red) in aortic sinus plaques in a progressive (ldlr−/− mice fed a 8-week HFD) or regressive (ldlr−/− mice fed a 8-week HFD and then switched to 4-week chow diet) environment. **b** Immunofluorescent staining of iNOS (M1 subtype macrophage marker, red) or Arg-1 (M2 subtype macrophage marker, red) in aortic sinus plaques both in progressive and regressive context. **c** Quantification of MDL-1 positive macrophages in **a**. **d** Real-time PCR analysis of MDL-1 mRNA in bone marrow-derived macrophages (BMDMs) polarized toward M1, M2, or unpolarized (M0). **e** Western-blot analysis of aortic lysates from apoE−/− mice fed a 8-week HFD or chow diet. All images are representative of at least three mice per group. Values are expressed as mean ± SEM. *p < 0.05 compared with baseline group mice or unpolarized BMDMs

To understand the dynamic changes of macrophage MDL-1 expression in atherosclerosis, we used an established model of plaque regression where ldlr−/− mice fed a 8-week HFD were either sacrificed for baseline lesion measurements or switched to a chow diet for another 4 weeks. This switch to chow is associated with significant reductions in total serum cholesterol and LDL cholesterol (all *p* < 0.05; Fig. 2). We observed that macrophage MDL-1 expression was remarkably decreased accompanied with reduced MOMA-2 positive macrophage content and iNOS expression in regressive plaques, which suggested decreased MDL-1 expression might be related to reduced number of lesional macrophages in regressive plaques and this is possibly caused by cell apoptosis process in early atherosclerotic lesion (*p* < 0.05; Fig. 3a–c). Besides, we also found an increased Arg-1 expression in regressive plaques, a marker of M2 subtype macrophages (Fig. 3b). Furthermore, to investigate whether MDL-1 expression is related to inflammatory lesional macrophage phenotype, we both measured MDL-1 expression in BMDM polarized in vitro and costained iNOS and MDL-1 in advanced and regressive atherosclerotic plaques in vivo. As what we hypothesized, the mRNA level of MDL-1 was significantly elevated in M1 subtype macrophages (*p* < 0.05; Fig. 3d), much higher than M2 subtype macrophages, and MDL-1 protein expression notably colocalized with iNOS in early advanced atheromata from baseline group (Additional file 3: Figure S3), which reflected its proinflammatory role in disease progression. Considering MDL-1 also expressed in neutrophil and neutrophil being an important contributor to plaque progression, we costained MDL-1 and neutrophil both in baseline and switch diet groups. Although MDL-1 expression was observed in a few lesional neutrophils, however, no significant difference was found between these two groups (Additional file 4: Figure S4).

In another established atherosclerotic model, apoE−/− mice were either fed a HFD or chow diet for 8 weeks and the aortic tissues were obtained after sacrifice. Notably, MDL-1 was abundantly expressed in aortic tissues of HFD-fed group mice compared with chow diet group, which suggested MDL-1 level increases in an early progressive environment such as hypercholesterolemic context (Fig. 3e).

### MDL-1 expression is regulated by pathophysiological agents implicated in atherosclerotic progression in vitro

**Fig. 4:**
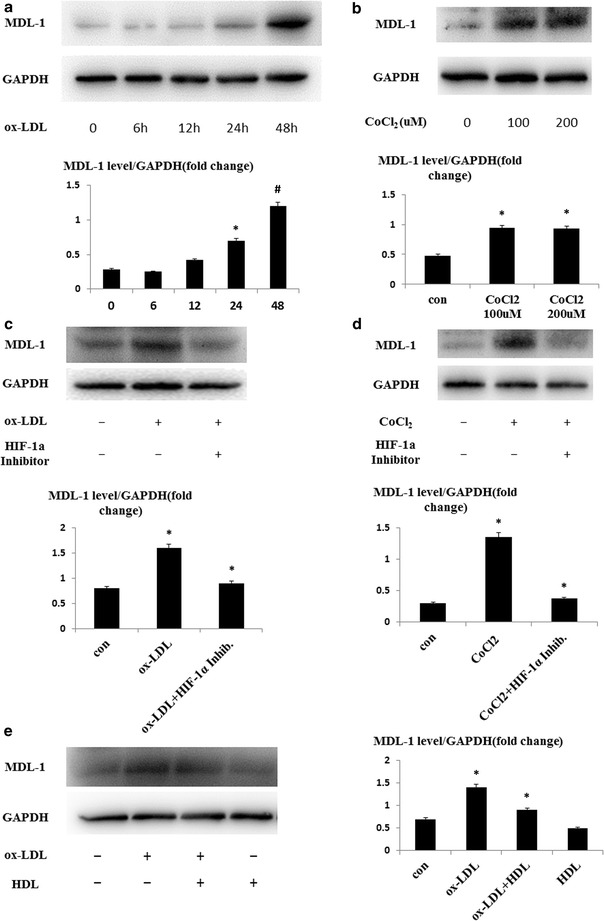
MDL-1 expression is regulated by pathophysiological agents implicated in atherosclerotic progression and regression in vitro. Western blot analyses of MDL-1 protein level in C57BL/6 mice peritoneal macrophages treated with **a** 50 μg/mL ox-LDL for the indicated times or **b** CoCl_2_ at the concentrations indicated for 24 h. Band densitometry are quantified. **c** Macrophages were stimulated with 50 μg/mL ox-LDL with or without pretreatment with 100 μmol/L HIF-1α inhibitor. **d** Macrophages were stimulated with 200 μmol/L CoCl_2_ with or without pretreatment with 100 μmol/L HIF-1α inhibitor. Band densitometry quantification are shown. **e** Macrophages were treated with 50 μg/mL ox-LDL with or without pretreatment with 10 μg/mL HDL. Images are representative of three independent experiments. Values are expressed as mean ± SEM. *p < 0.05; ^#^ *p* < 0.01

To further investigate molecular mechanisms which affected MDL-1 expression in the progression and regression of atherosclerosis and demonstrate what we observed in mouse models and polarized BMDMs, we used several pathophysiological agents implicated in plaque progression (i.e. ox-LDL and hypoxia) or regression (i.e. HDL) to stimulate macrophages and detected MDL-1 expression in vitro [26, 27]. Remarkably, we found MDL-1 expression was upregulated after ox-LDL stimulation for 24 h and more significant than baseline after 48 h (all *p* < 0.05; Fig. 4a). However, native LDL did not cause this alteration of MDL-1 expression (data not shown). Because ox-LDL induces plaque oxidative stress and its downstream transcription factor HIF-1α, we pretreated macrophages with HIF-1α inhibitor which could block its downstream transcription factor activity. Notably, we found ox-LDL-caused elevation of MDL-1 expression was blocked after pretreatment with HIF-1α inhibitor (all *p* < 0.05; Fig. 4c). Since hypoxia and HIF-1α have also been demonstrated in promoting atherosclerotic progression [28, 29], we then treated peritoneal macrophages with a chemical mimic of hypoxia, CoCl_2_, which can stabilize the downstream transcriptional factor HIF-1α. Similarly, we found CoCl_2_ stimulation remarkably induced MDL-1 expression and the increase of MDL-1 expression was also inhibited after pretreatment with HIF-1α inhibitor (all *p* < 0.05; Fig. 4b, d).

Moreover, we observed that the increase of MDL-1 expression was partly reversed after treatment of HDL, a lipoprotein implicated in promotion of cholesterol efflux from the cells and blocks inflammatory effects of ox-LDL in atherosclerotic regression (all *p* < 0.05; Fig. 4e) [30]. Whereas, treatment with HDL alone did not cause any change of MDL-1 level. These results reflect the dynamic changes of macrophage MDL-1 expression regulated by both pro- and anti-atherosclerotic stimuli.

### MDL-1 overexpression protects macrophages from Ox-LDL induced apoptosis in vitro

**Fig. 5:**
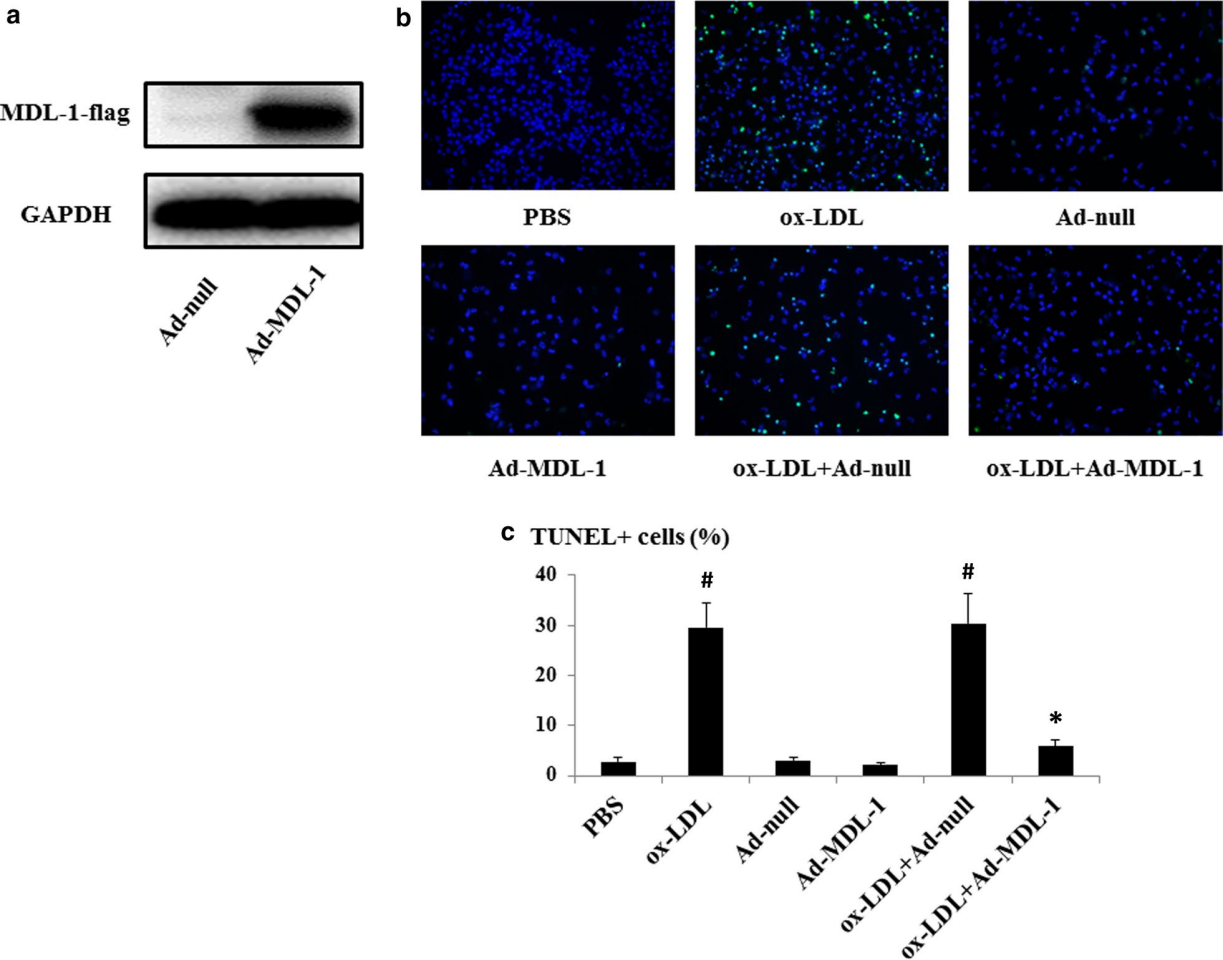
MDL-1 overexpression promotes macrophage survival under ox-LDL stimulation. **a** Western-blot analysis of MDL-1 expression in peritoneal macrophages after infected with adenovirus overexpressing MDL-1 (Ad-MDL-1) or control virus (Ad-null). **b** Macrophage apoptosis (green) measured by TUNEL assay of peritoneal macrophages stimulated with 50 μg/mL ox-LDL with or without pretreatment with Ad-MDL-1 or Ad-null in RPMI 1640 media. Nuclei were costained with DAPI (blue). Quantification of tunel assay from each group (n ≥ 3/group) was performed in (**c**). Values are expressed as mean ± SEM. *p < 0.05 compared with ox-LDL with Ad-null treatment group; ^#^ *p* < 0.01 compared with PBS or Ad-null group

Previous research on mouse myeloid lineage cells found MDL-1 stimulation helps to maintain 32Dcl3 cell survival [10]. Therefore, we detected whether increased MDL-1 expression could affect macrophage apoptosis under atherogenic stimuli (e.g. ox-LDL). Here, we used a MDL-1 overexpressed adenovirus, to infect peritoneal macrophages before exposed to ox-LDL (Fig. 5a). In TUNEL assay, primary macrophages underwent apoptotic process after high-dose ox-LDL stimulation. Surprisingly, these macrophages were largely protected from apoptosis when pretreated with adenovirus overexpressing MDL-1 (Ad-MDL-1) compared with control virus (all *p* < 0.05; Fig. 5b, c). However, treatment with Ad-MDL-1 alone did not exhibit any difference compared with control group (Fig. 5b, c).

**Fig. 6:**
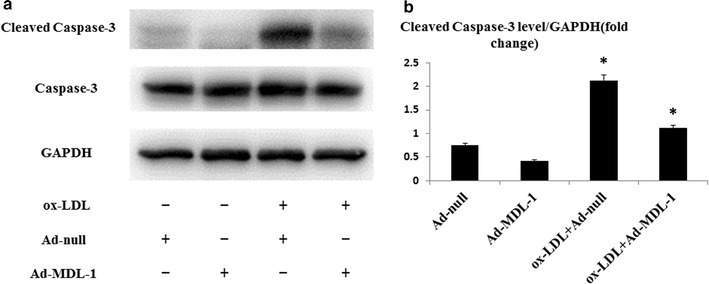
MDL-1 overexpression reduces apoptosis-associated molecule expression in macrophages under ox-LDL stimulation. **a** Representative Western blot analyses of cleaved caspase-3 and caspase-3 expression in cell lysates from peritoneal macrophages treated with 50 μg/mL ox-LDL with or without pretreatment of adenovirus overexpressing MDL-1 (Ad-MDL-1) or control virus (Ad-null) in RPMI 1640 media. Images are representative of three independent experiments. Quantification of band densitometry is shown in **b**. Values are expressed as mean ± SEM. *p < 0.05 compared with Ad-null or ox-LDL with Ad-null treatment group

Furthermore, we next detected active cleaved caspase-3 expression in peritoneal macrophages, molecular mechanism associated with cell apoptosis, under ox-LDL stimulation. As what we expected, the level of cleaved caspase-3 was significantly elevated after ox-LDL stimulation and this increased expression was notably inhibited in Ad-MDL-1 infected macrophages (all *p* < 0.05; Fig. 6a, b), which was coincident with the results of TUNEL assay. Moreover, these Ad-MDL-1 infected macrophages induced more MCP-1 production under ox-LDL stimulation compared with control virus group (Additional file 5: Figure S5), which manifested the survival macrophages can cause more macrophage migration and accumulation. These results indicate that increased expression of MDL-1 promotes macrophages survival under atherogenic stimuli, which could be predicted to foster lesional macrophage accumulation and increase foam cell burden during early atherosclerosis progression.

## Discussion

Atherosclerosis progression is characterized by persistent accumulation of lipids and immune cells in plaques, which is the leading cause of atherosclerotic plaque rupture and contributes to myocardial infarction or other adverse cardiovascular outcomes. Rupture-prone plaques feature with a thin fibrous cap and a large lipid necrotic core [31]. Lesional macrophages are central in these pathophysiological processes and recent studies suggest macrophage appears in a dynamic balance in the lesion and strategies aiming at reducing macrophage-derived foam cell burden could significantly promotes atherosclerotic plaque regression and stability [3, 32, 33]. However, the mechanisms in lesional macrophage retention and accumulation are, to a large extent, still elusive and unknown. Our study firstly demonstrated that MDL-1, a C-type lectin receptor, is mainly expressed in lesional macrophages and its expression is associated with early atherosclerotic plaque progression both in two well-established models (apoE−/− and ldlr−/− mice). Furthermore, we observed macrophage MDL-1 expression is notably decreased accompanied with reduced lesional macrophage content and M1 subtype macrophage marker iNOS expression in plaque regression group mice and it is significantly expressed in polarized M1 macrophage, reflecting its proinflammatory role in the disease. Moreover, our in vitro experiments found MDL-1 expression is driven by several pathophysiological agents implicated in plaque progression (e.g. ox-LDL and hypoxia) and the increased MDL-1 expression can be blocked by regressive agents like HDL and HIF-1α inhibitor. In addition, we found MDL-1 overexpression could maintain macrophage survival, downregulate cleaved caspase-3 expression and induce MCP-1 production under atherosclerotic agents stimulation (e.g. ox-LDL). Therefore, MDL-1 is predicted to cause inflammatory lesional macrophage accumulation in the context of early atherosclerosis and these discoveries provide a novel macrophage receptor biomarker regarding to the relationship between its level and atherosclerotic plaque progression and link abnormal lipid level to macrophage survival and accumulation, which might further aggravate plaque foam cell burden and promote atherosclerosis progression.

MDL-1, also called CLEC5A, associates non-covalently with its adaptor DAP-12 to form receptor complexes and mediate its signaling pathway [8]. Several previous studies showed that MDL-1 is mainly expressed in mouse neutrophils, monocytes/macrophages and human peripheral blood monocytes as well as myeloid cell precursors [9–11, 18]. In addition, stronger MDL-1 expression was observed in mature differentiated myelomonocytic cells [11]. These results were coincident with our finding that MDL-1 is highly expressed in lesional macrophages in both human and apoE−/− mice advanced atheromata and MDL-1 colocalized with DAP-12 in atherosclerotic plaques. Besides, we also observed some MDL-1 positive cells were not macrophage-derived. Considering numerous related studies described above and neutrophil also being an important contributor to plaque progression [32], these cells might come from neutrophils. This conclusion was also demonstrated in our immunofluorescent costaining of ldlr−/− mice lesional neutrophil and MDL-1, which MDL-1 was found to be expressed in a few of neutrophils. However, we did not find significant difference on neutrophil MDL-1 expression between progressive and regressive plaques. This may attribute to different MDL-1 expression pattern in neutrophil and the extent of neutrophil MDL-1 expression is more relevant to cell differentiation [10]. Functionally, MDL-1 has been increasingly recognized as a key regulator involving in the progression of multiple acute and chronic inflammatory diseases including hemorrhagic fever, lethal shock, virus-induced brain damage, chronic obstructive pulmonary disease and the development of autoimmune arthritis [13, 15–18]. Furthermore, MDL-1 promotes proinflammatory cytokines secretion and macrophage activation in these inflammation-associated diseases [12, 34]. In inflammatory macrophage, activation of Syk-coupled MDL-1 induces transcription of pro-interleukin-1β (IL-1β) and NLRP-3 inflammasome during DV infection [35]. These functions of MDL-1 might partly explain what we have observed in this study, which significant increased MDL-1 expression is found on M1 proinflammatory macrophage subtype, PBMCs from significant CAD patients and associated with inflammatory chemokine (e.g. MCP-1) production and atherosclerotic plaque progression.

Our in vitro experiments exhibited that MDL-1 expression in protein level is driven by several atherosclerotic pathophysiological agents like ox-LDL and hypoxia. We also found macrophage MDL-1 expression were not induced by native LDL. As we know, ox-LDL but not LDL has been described as potential promoter of vascular inflammatory responses and oxidative stress during atherosclerosis [36]. And MDL-1, a proinflammatory receptor, were found in M1 subtype macrophages and could be upregulated by hypoxia in our study. Besides, we found that increased MDL-1 expression is blocked after HDL or HIF-1α inhibitor pretreatment. Previous studies have demonstrated that hypoxia exists in lipid enriched advanced plaques and it is coexpressed with netrin-1 [28], one of the neuronal guidance cues implicated in macrophage retention and leading to foam cell accumulation [37]. Investigation also showed that HIF-1α, a downstream transcription factor of ox-LDL, could affect lesional macrophage inflammatory profile and promote development of atherosclerosis [29]. Therefore, increased MDL-1 expression induced by ox-LDL or hypoxia and inhibited by HDL or HIF-1α inhibitor suggests that MDL-1 might participate in lesional macrophage accumulation and the failure to resolve inflammation under proatherogenic conditions. This conclusion is further supported when we found that MDL-1 expression is downregulated in lesional macrophages in a regressive plaque milieu accompanied with a reduction in lesional macrophage content. And as what we observed in regressive plaque, this reduced expression of MDL-1 might be due to decreased number of lesional macrophages.

Since our study indicated that changes of macrophage MDL-1 expression also accompanied with alterations of lesional macrophage content, we conducted the TUNEL assay and found that overexpression of MDL-1 contributed to significant decrease of macrophage apoptosis under high-dose ox-LDL stimulation. This phenomenon is also in accordance with the finding that MDL-1 stimulation helps to maintain 32Dcl3 cell survival [10]. Although our results showed increased MDL-1 expression can be induced by ox-LDL, we here used an high-dose extensively oxidized LDL to stimulate the cells for 24 h and previous investigations demonstrated low-dose minimally oxidized LDL could lead to macrophage survival [38]. Past research has demonstrated that local apoptosis mainly mediates clearance of macrophages from resolving inflammation and controls macrophage removal [39]. As we know, in the early stage of atherosclerotic lesions, increased resistance of macrophages to apoptosis is associated with macrophage accumulation and increased plaque burden [40]. This conclusion is also coincident with the results of studies which revealed that apoptotic cells are only detectable in advanced lesions and this reflects an efficient apoptosis clearance mechanism (e.g. normal efferocytosis function) in the early stage of atherosclerotic plaques and any disruption of macrophage physiological apoptotic process in this stage may contribute to lesion progression [6, 41, 42]. Furthermore, we also found MDL-1 overexpression in these macrophages amplified ox-LDL induced MCP-1 production. Therefore, the results of this study, which indicated MDL-1 overexpression promotes macrophage survival and subsequently amplifies chemokine production, suggest MDL-1, a receptor involving in multiple acute and chronic inflammatory diseases, is associated with early atherosclerotic plaque progression.

## Conclusions

MDL-1 is abundantly expressed in macrophages of human and apoE−/− as well as ldlr−/− mice advanced atherosclerotic lesions and increased macrophage MDL-1 expression is associated with early plaque progression and promotes macrophage survival under proatherosclerotic stimuli (e.g. ox-LDL), thereby implicating that it might maintain lesional macrophage survival and link abnormal hyperlipidemic environment to macrophage accumulation during early atherosclerotic plaque progression. Our study indicated MDL-1 may be developed as a potent biomarker for predicting and preventing early atherosclerotic plaque progression.

## Additional files

**Additional file 1: Figure S1.** MDL-1 and its adaptor DAP-12 colocalize in advanced apoE−/− mice atherosclerotic plaques. Immunostaining of MDL-1 (green) and DAP-12 (red) in apoE−/− mice advanced atherosclerotic plaques. Areas of colocalization are shown in yellow in the merged image (shown with white arrows). Images are representative of 3 mice.

**Additional file 2: Figure S2.** Increased MDL-1 level in human peripheral blood monocytes is associated with the presence of coronary artery disease (CAD). Western-blot analyses of MDL-1 protein expression in peripheral blood monocytes from significant CAD patients (n = 3) and healthy volunteers (n = 3). Band densitometry are quantified. Values are expressed as mean ± SEM. ^#^ *p* < 0.01 compared with healthy volunteers.

**Additional file 3: Figure S3.** MDL-1 and M1 subtype macrophage marker iNOS colocalize in early advanced ldlr−/− mice atherosclerotic lesion and both reduce in regressive plaque. Immunofluorescent costaining of MDL-1 (green) and iNOS (red) in aortic sinus plaques in a progressive (ldlr−/− mice fed a 8-week HFD) or regressive (ldlr−/− mice fed a 8-week HFD and then switched to 4-week chow diet) environment. Areas of colocalization are exhibited in yellow in the merged image. Images are representative of at least 3 mice.

**Additional file 4: Figure S4.** No significant difference is found on neutrophil MDL-1 expression between progressive and regressive atherosclerotic plaques. MDL-1 (green) and Ly-6G (red) were costained in ldlr−/− mice advanced or regressive atherosclerotic plaques. Areas of colocalization are exhibited in yellow in the merged image (shown with white arrows). Images are representative of at least 3 mice.

**Additional file 5: Figure S5.** MDL-1 overexpression amplifies ox-LDL induced monocyte chemotactic protein 1 (MCP-1) production in peritoneal primary macrophages. Representative Western blot analyses of MCP-1 expression in cell lysates from peritoneal macrophages treated with 50 μg/mL ox-LDL with or without pretreatment of adenovirus overexpressing MDL-1 (Ad-MDL-1) or control virus (Ad-null) in RPMI 1640 media. Images are representative of 3 independent experiments.

## Abbreviations

MDL-1: myeloid DAP12-associating lectin-1
CLEC5A: C-type lectin domain family 5-member A
ox-LDL: oxidized low-density lipoprotein
ITAM: immuno-receptor tyrosine-based activation motif
CAD: coronary artery disease
CCL: chemokine (C–C motif) ligand
HFD: high-fat diet
HDL: high-density lipoprotein
M-CSF: monocyte-colony stimulating factor
BMDMs: bone marrow-derived macrophages
FBS: fetal bovine serum
PBS: phosphate buffered saline
IL: interleukin
LPS: lipopolysaccharide
EDTA: ethylene diamine tetraacetic acid
TC: total cholesterol
LDL/VLDL: low-density lipoprotein/very low-density lipoprotein
WD: western diet
MCP-1: monocyte chemotactic protein 1
