# Supplementary figures and images for: The macrophage C-type lectin receptor CLEC5A (MDL-1) expression is associated with early plaque progression and promotes macrophage survival
Source: J Transl Med. 2017 Nov 10;15:234. doi: 10.1186/s12967-017-1336-z (PMC5681784; doi:10.1186/s12967-017-1336-z)

## Slide 1
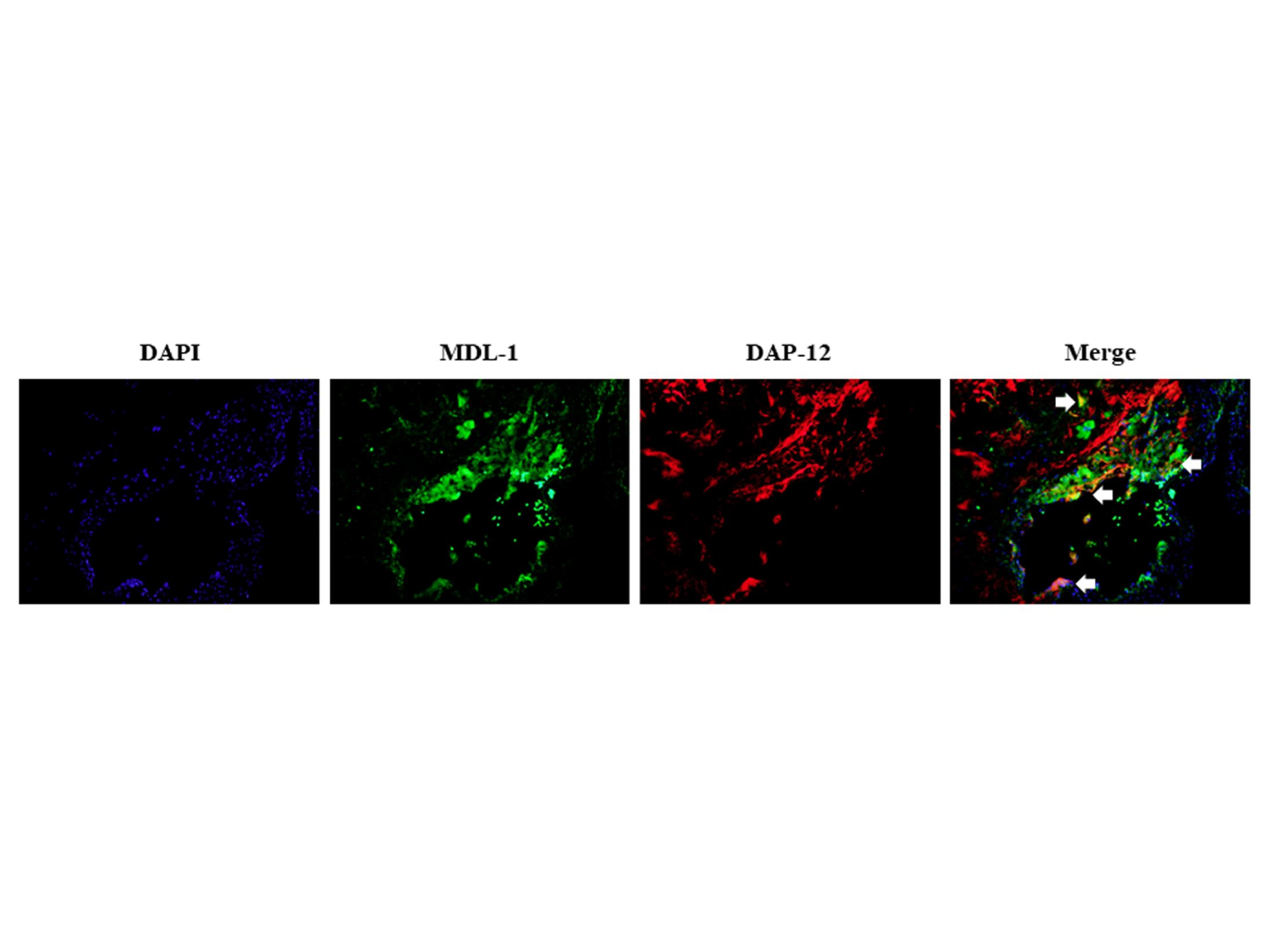

Supplement: Supplementary file 1 — Additional file 1: Figure S1. MDL-1 and its adaptor DAP-12 colocalize in advanced apoE−/− mice atherosclerotic plaques. Immunostaining of MDL-1 (green) and DAP-12 (red) in apoE−/− mice advanced atherosclerotic plaques. Areas of colocalization are shown in yellow in the merged image (shown with white arrows). Images are representative of 3 mice. [file 12967_2017_1336_MOESM1_ESM.pptx]

## Slide 1
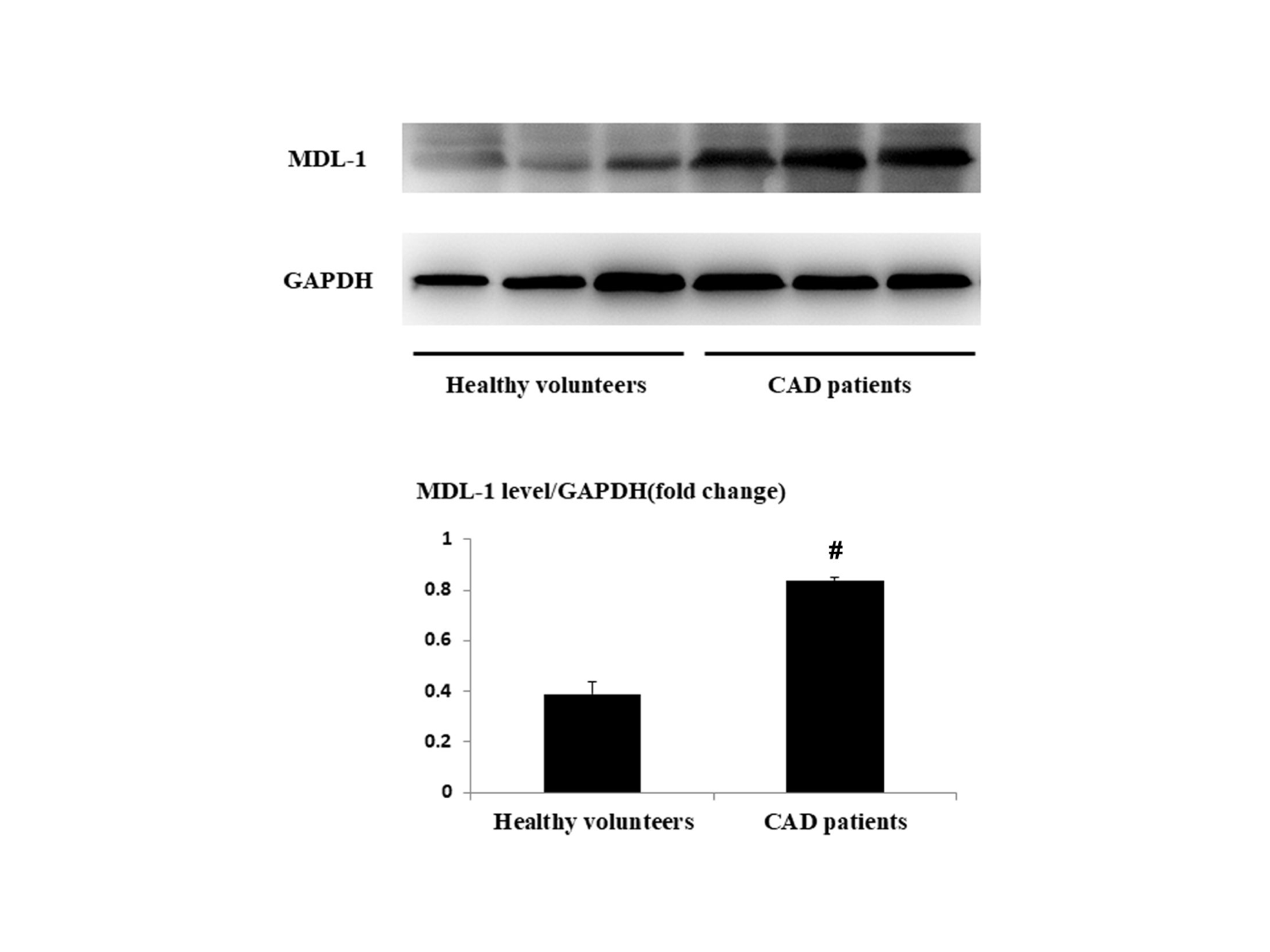

Supplement: Supplementary file 2 — Additional file 2: Figure S2. Increased MDL-1 level in human peripheral blood monocytes is associated with the presence of coronary artery disease (CAD). Western-blot analyses of MDL-1 protein expression in peripheral blood monocytes from significant CAD patients (n = 3) and healthy volunteers (n = 3). Band densitometry are quantified. Values are expressed as mean ± SEM. # p < 0.01 compared with healthy volunteers. [file 12967_2017_1336_MOESM2_ESM.pptx]

## Slide 1
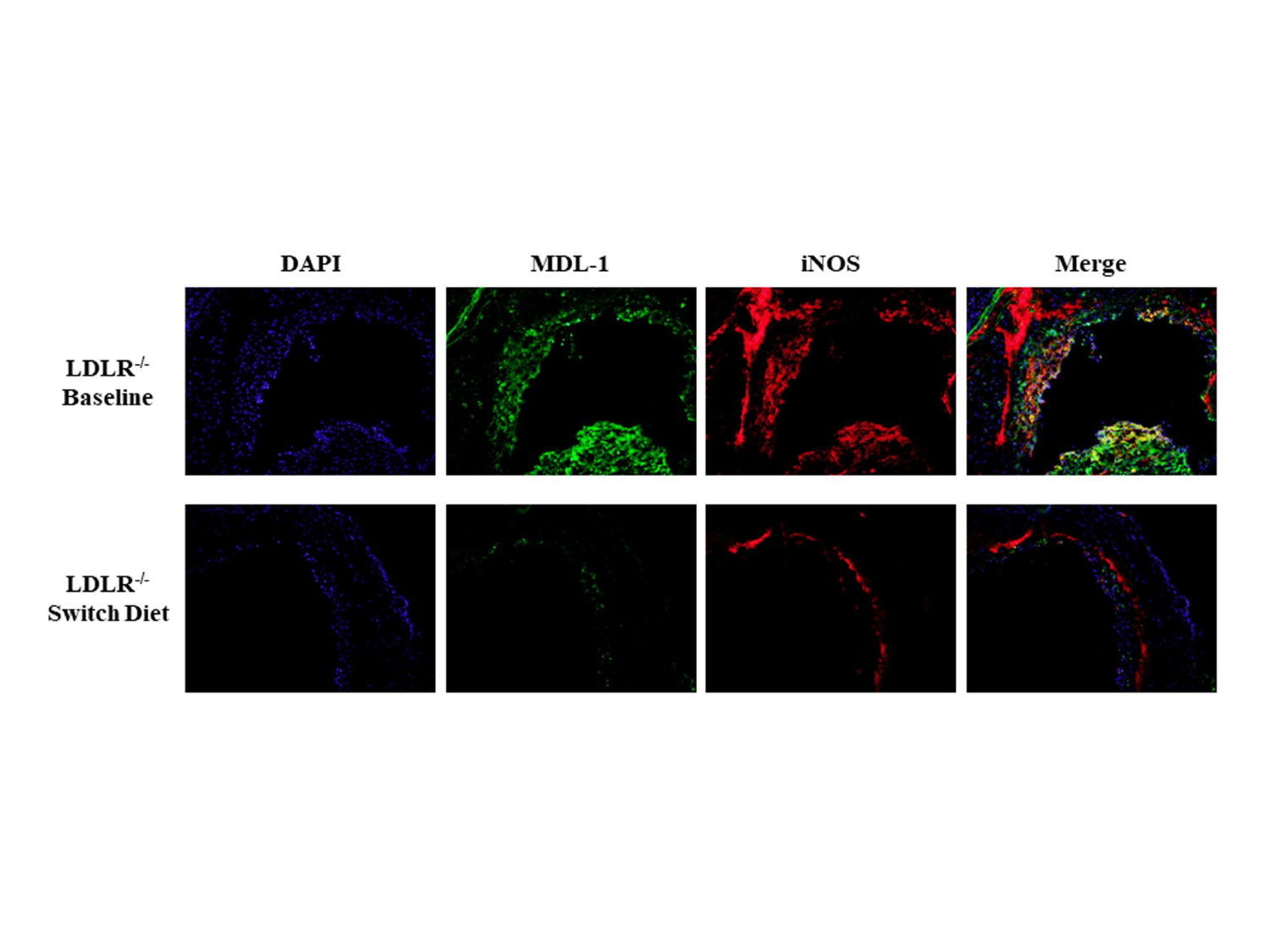

Supplement: Supplementary file 3 — Additional file 3: Figure S3. MDL-1 and M1 subtype macrophage marker iNOS colocalize in early advanced ldlr−/− mice atherosclerotic lesion and both reduce in regressive plaque. Immunofluorescent costaining of MDL-1 (green) and iNOS (red) in aortic sinus plaques in a progressive (ldlr−/− mice fed a 8-week HFD) or regressive (ldlr−/− mice fed a 8-week HFD and then switched to 4-week chow diet) environment. Areas of colocalization are exhibited in yellow in the merged image. Images are representative of at least 3 mice. [file 12967_2017_1336_MOESM3_ESM.pptx]

## Slide 1
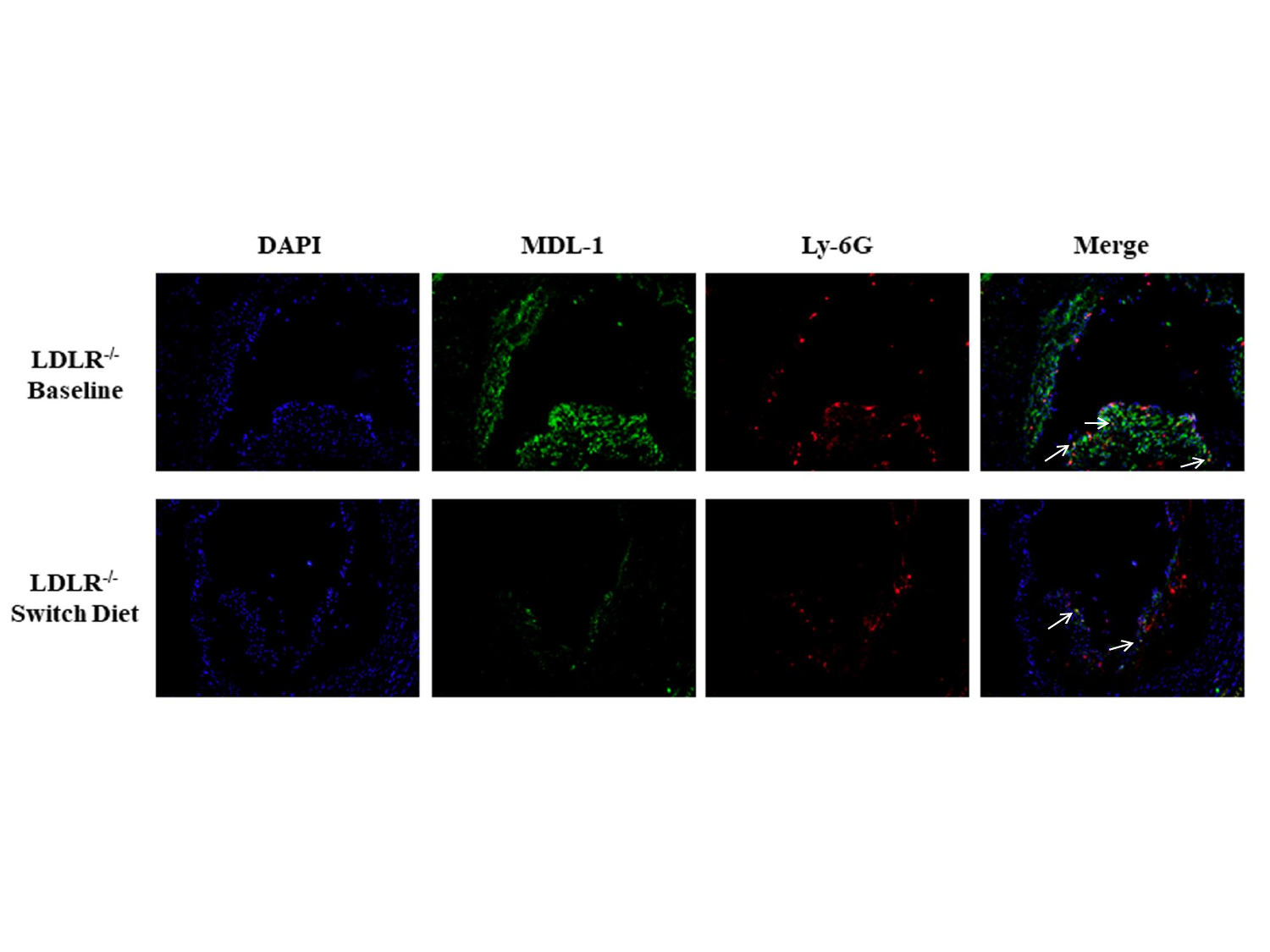

Supplement: Supplementary file 4 — Additional file 4: Figure S4. No significant difference is found on neutrophil MDL-1 expression between progressive and regressive atherosclerotic plaques. MDL-1 (green) and Ly-6G (red) were costained in ldlr−/− mice advanced or regressive atherosclerotic plaques. Areas of colocalization are exhibited in yellow in the merged image (shown with white arrows). Images are representative of at least 3 mice. [file 12967_2017_1336_MOESM4_ESM.pptx]

## Slide 1
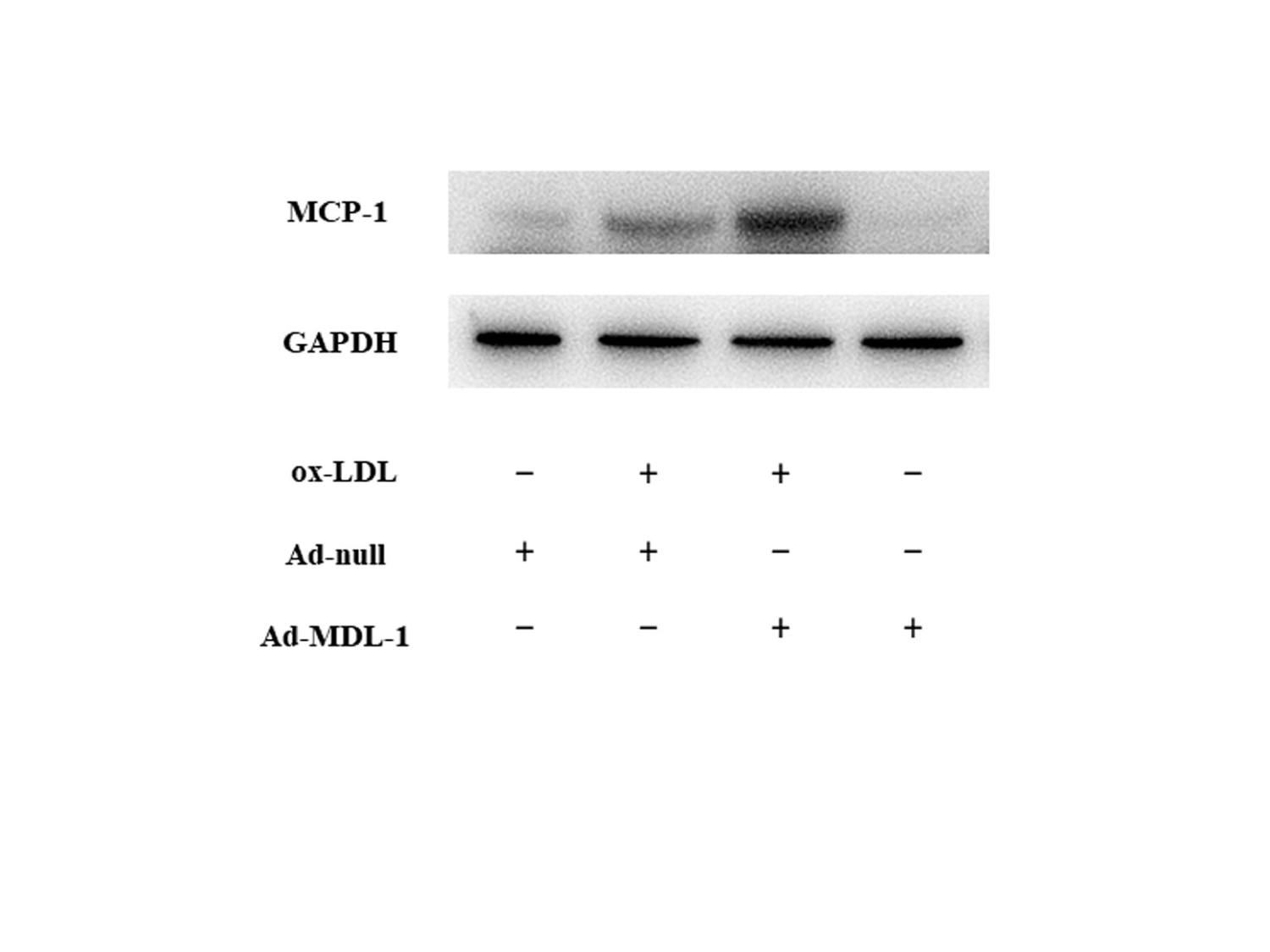

Supplement: Supplementary file 5 — Additional file 5: Figure S5. MDL-1 overexpression amplifies ox-LDL induced monocyte chemotactic protein 1 (MCP-1) production in peritoneal primary macrophages. Representative Western blot analyses of MCP-1 expression in cell lysates from peritoneal macrophages treated with 50 μg/mL ox-LDL with or without pretreatment of adenovirus overexpressing MDL-1 (Ad-MDL-1) or control virus (Ad-null) in RPMI 1640 media. Images are representative of 3 independent experiments. [file 12967_2017_1336_MOESM5_ESM.pptx]
